# Plasma cell leukemia presenting as spontaneous tumor lysis syndrome with hypercalcemia

**DOI:** 10.1002/ccr3.5933

**Published:** 2022-07-14

**Authors:** Mahan Shafie, Mahbod Issaiy, Mahdi Barkhori, Samaneh Parsa

**Affiliations:** ^1^ 48439 School of Medicine Tehran University of Medical Sciences Tehran Iran; ^2^ 48439 Department of Internal Medicine Imam Khomeini Hospital Complex Tehran University of Medical Sciences Tehran Iran

**Keywords:** calcium, hypercalcemia, leukemia, plasma cell leukemia, spontaneous tumor lysis syndrome, tumor lysis syndrome

## Abstract

Tumor lysis syndrome (TLS) is an oncologic emergency in which tumor cells undergo lysis either spontaneously or due to the initiation of cancer therapy typically presenting with hypocalcemia. We described a 62‐year‐old male patient with spontaneous TLS and hypercalcemia without a known malignancy, who is later discovered to have plasma cell leukemia.

## Abstract 2

A high level of suspicion is needed for spontaneous tumor lysis syndrome to be diagnosed in early stages to provide a better chance of more immediate and effective treatments, especially in invasive malignancies such as plasma cell leukemia.

## 1 INTRODUCTION

Tumor lysis syndrome (TLS) is an oncologic emergency in which tumor cells undergo lysis either spontaneously or due to the initiation of cancer therapy.^1^ It predominantly occurs in the setting of hematological malignancies, particularly non‐Hodgkin’s lymphoma and acute leukemia.^2^ It typically manifests with biochemical abnormalities such as hyperkalemia, hyperphosphatemia, hyperuricemia, and hypocalcemia.^1^, ^2^ Plasma cell leukemia (PCL) is an uncommon and aggressive variant of multiple myeloma with poor survival albeit with a clinically and genetically distinct profile.^3^, ^4^ In the following case report, we present a 62‐year‐old male patient with spontaneous TLS and hypercalcemia without a known malignancy, who is later discovered to have PCL.

## 2 CASE PRESENTATION

The patient was a 62‐year‐old man with a past medical history of hypertension and a habitual history of cigarette smoking and methadone consumption. He presented to the emergency department with nausea, vomiting, diarrhea, pruritus, and malaise starting from 4 to 5 days prior to presentation. On initial examination, he was suffering from jaundice and non‐pitting bilateral edema involving his feet and calves. He had asterixis and was confused when performing neurological examinations. Vital signs were within normal range. The initial laboratory tests revealed severe leukocytosis, anemia, thrombocytopenia, high creatinine and blood urea nitrogen levels, increased liver enzymes, elevated serum calcium and phosphorus levels, and a high D‐Dimer titer (Table 1). Ultrasonography of abdomen and pelvis showed dilated common bile duct, increased echogenicity of parenchyma, and increased corticomedullary distinction of the kidneys. The patient's ECG showed normal sinus rhythm. Due to the signs of encephalopathy and his hematologic state, he underwent emergent hemodialysis and received packed red blood cells and platelets. The patient's echocardiography results revealed bilateral atrial enlargement, mild left ventricular enlargement and mild systolic dysfunction, mild to moderate right ventricular enlargement and systolic dysfunction, global hypokinesia, mild mitral regurgitation, moderate tricuspid regurgitation, a pulmonary artery pressure of 37 mmHg, and ejection fraction of 45%. After being transferred to ward, he was hemodialyzed regularly and supportive therapy was started focusing on his kidney function, serum electrolyte balance, and hematologic state. Due to his volume overload, we initiated furosemide. In order to treat the patient's electrolyte imbalances (hypercalcemia, hyperphosphatemia, and hyperuricemia) after intravenous fluid replacement as a first step of the management, we administered pamidronate, calcitonin, sevelamer, and rasburicase (after confirming sufficient levels of G6PD). Dexamethasone was ordered because multiple myeloma was suspected as the underlying cause since it may produce a similar clinical profile with hypercalcemia. Adequate hydration was provided via crystalloid fluids, mainly isotonic saline (0.9% NaCl). The patient's conditions raised the suspicion for TLS; therefore, hematology service was consulted and they requested flow‐cytometry, bone marrow biopsy, and aspiration for a more accurate diagnosis. During the course of his hospitalization, the patient's signs and symptoms were improving and his laboratory tests were stabilizing. However, on the 14th day of hospitalization, he experienced respiratory distress, and on the 15th day, he unfortunately passed away due to cardiac arrythmia and asystole. After the patient's decease, the results of the flow‐cytometry test and peripheral blood smear (PBS) arrived. PBS examination showed more than 20% mononuclear cells in the smear that which resembled plasma cells. Flow‐cytometry revealed a population in monocytic gate (about 37%) that were positive for CD38, CD138, CD56, and negative for CD45, CD19 (myeloma cells). The findings are concordant with PCL.

**TABLE 1:** Laboratory data of the patient during hospitalization

	Laboratory/Day of hospitalization	0th	1st	2nd	3rd	4th	5th	7th	8th	9th	10th	Unit	Reference range
CBC	WBC	54.2	53.5		43.8			34.5	34.3			×1000/mm^3^	4.1–10.1
	Hgb	6.3	5.6		7.7			8.4	8.4			g/dl	12–16
	MCV	131	135		118			117	114			fl	77–94
	PLT	37	36		42			34	45			×1000/mm^3^	150–400
	Retic (%)							6.1				%	0.5–1.5
Electrolytes	Na				136	136	135	131	130	141	137	mEq/L	135–145
	K			4.7	5	4.6	4.6	4.8	4.6	4.8	4.5	mEq/L	3.5–5
	Ca			13.7	12.6	9.6	9	8.6	8.6	8.7	8.2	mg/dl	8.6–10.2
	P				14.7	9.2	9.8	10	10.7	10.1	10.4	mg/dl	2.5–5
	Mg				2.5	2.2	1.8	2.2	2.5	2.5	2.5	mg/dl	1.6–2.6
	Cr	7.8		7.9	6.4	4.8	4.9	3.7	4.4	4.2	4.5	mg/dl	0.7–1.4
	BUN	110										mg/dl	7–21
	Urea				86	68		79	103	93	122	mg/dl	15–50
	Uric acid				8.3	5.8		1.9	3.3	3.6	5.3	mg/dl	3.6–8.2
	PTH	68										pg/ml	10–69
Thyroid hormones	TSH	4.21											
	Free T4	0.93										mIU/L	0.5–5.0
	T3	1.47										ng/dl	0.7–1.9
	Blood culture			24h: neg								nmol/L	0.9–2.8
Prostate tumor markers	PSA total	0.29										ng/ml	
	PSA free	0.13										ng/ml	
	Pro‐calcitonin								<0.2			ng/ml	<0.1
	G6PD					Sufficient							
	LDH						809					U/L	140–280
	Folic acid			6								ng/ml	2.7–17
	B12			191								pg/ml	190–950
	Vitamin D3 (25OH)	16.7										ng/dl	25–80
Coagulation	D‐dimer			6181			>10,000					ng/ml	<250
	Fibrinogen			210								mg/dl	200–400
VBG	pH			7.35									7.35–7.45
	pCO_2_			46								mmHg	35–45
	HCO_3_			26								mmol/L	22–28
Liver enzymes	AST	71										U/L	10–40
	ALT	65										U/L	29–33
	ALP	611										IU/L	30–120
Urine analysis	Protein				3+								
	Glucose				neg								
	Blood				1+								
	WBC				2–4							cells/HPF	2–5
	RBC				8–10							cells/HPF	0–4
	Bac				neg								
	Crystal				urate amorph: many								
	Culture				neg								
Serology brucellosis	Wright	Neg											
	2ME	Neg											
	Direct Coombs	Neg											
Viral markers	HBsAg								non‐reactive				
	Anti‐HCV								non‐reactive				
	HIV Ag/Ab								non‐reactive				

Abbreviations: 2ME, 2‐mercaptiethanol brucella agglutination test; ALT, alanine transaminase; AST, aspartate transaminase; BUN, blood urea nitrogen; Cr, creatinine; G6PD, glucose‐6‐phosphate dehydrogenase; Hgb, hemoglobin; LDH, lactate dehydrogenase; MCV, mean corpuscular volume; PLT, platelet; PSA, prostate‐specific antigen; PTH, parathyroid hormone; TSH, thyroid stimulating hormone; WBC, white blood cell.

## 3 DISCUSSION

We presented a fatal case of spontaneous tumor lysis syndrome (STLS) and hypercalcemia in a patient with plasma cell leukemia (PCL). Tumor lysis syndrome is a critical oncologic emergency usually occurs after chemotherapy or any other treatment of a malignancy, resulting the release of intracellular contents into the extracellular compartment. TLS usually leads to a constellation of metabolic abnormalities including hyperuricemia, hyperphosphatemia, hyperkalemia, and hypocalcemia.^5^ STLS is the development of TLS in patients with malignancy without receiving any specific treatment for cancer. STLS occurs mostly in hematologic malignancies, and according to previous studies, it has been suggested that STLS is associated with a high mortality rate.^6^

The diagnosis of TLS is based on the Cairo–Bishop criteria, which include laboratory (hyperuricemia, hyperphosphatemia, hyperkalemia, and hypocalcemia) and clinical (Increased creatinine, cardiac arrhythmia/sudden death, and seizure) findings.^7^ Unlike most TLS cases, our patient presented hypercalcemia in initial laboratory tests instead of hypocalcemia, which could be related to nature of plasma cell dyscrasias, particularly in this case, primary PCL. In these hematologic malignancies, malignant cells produce substances that induce osteoclasts to accelerate the rate of bone break down. This increases the levels of calcium in the blood even in the absence of other etiologies of hypercalcemia in hematologic malignancies (i.e., ectopic production of PTH, high level of vitamin D, PTH‐rP production, or bone metastasis).^8^ Based on our literature review, there is not an exact investigation of the prevalence of hypercalcemia in TLS patients; however, there are a few reports of TLS cases paradoxically associated with hypercalcemia due to different etiological bases.^9^, ^10^, ^11^

Although the elevation of lactate dehydrogenase (LDH), which was detected in our patient, is not included in the laboratory definition of TLS, it is shown to be a very sensitive marker for TLS.^12^ PCL diagnosis requires plasma cell absolute count of at least 2 × 10^9^/L and 20% of the peripheral blood white cells,^13^ as was the case in our patient. Although, these criteria are under investigation and may change in the future. There are general and cancer‐related risk factors for the development of TLS. Burkitt's lymphoma/leukemia, acute lymphocytic leukemia, acute myeloid leukemia, diffuse large B‐cell lymphoma, and bulky tumors carry the highest risk of developing into TLS. However, multiple myeloma and other plasma cell dyscrasias, on the contrary, are considered low‐risk malignancies for TLS. General risk factors include old age, high LDH plasma levels, and baseline kidney disease, which were present in our patient.^12^

Tumor lysis syndrome is a serious oncologic emergency with a high mortality rate; hence, patients at risk of developing TLS should receive immediate and intensive treatment. Laboratory tests including serum electrolytes, uric acid, and creatinine should be carefully checked. Cardiac arrhythmia and sudden death can occur; so, cardiac monitoring should be performed, calcium gluconate may need to be administrated, and potassium‐lowering strategies such as glucose/insulin infusion, beta‐agonist inhalation, and oral potassium‐binding agents may also be used. Calcium treatment should not be prescribed in asymptomatic hypocalcemia due to risk of calcium phosphate formation in different tissues such as kidney and heart. Hypouricemic agents such as allopurinol and rasburicase can be helpful in lowering serum uric acid levels. If the above measures are not effective and the following conditions are met, hemodialysis will be indicated in a case with severe oliguria or anuria, persistent hyperkalemia, hyperphosphatemia induced symptomatic hypocalcemia, and calcium phosphate product ≥70 mg/dl.^14^

According to literature, a number of hematologic and solid malignancies have been reported developing STLS as a first presentation of the disease. Only one case of PCL developing into STLS has been reported so far. Kollathodi, et al.^15^ described a 28‐year‐old man with PCL that developed to STLS. The patient was initially suspected of having tuberculosis and treatment was started, but because he was not relieved, he was referred to another center for further investigation. Our literature review suggested that although STLS has a worse prognosis in some cases, due to the poor prognosis of TLS in malignancies, particularly STLS, it is essential to consider the fact that TLS can occur spontaneously and it is important to initiate immediate treatment as any suspicious symptoms are noticed.^14^ Meanwhile, PCL is an aggressive malignancy, and the overall prognosis of PCL is very poor with a median survival of 7–11 months, with up to 28% dying within the first month after diagnosis. It is even shorter when PCL occurs simultaneously with complications such as TLS. However, with better management of complications, and the widespread use of high‐dose therapy with autologous hematopoietic cell transplantation and other novel agents, the overall survival can be improved.^13^

In conclusion, TLS is a high‐mortality oncological emergency and specifically, STLS is often underdiagnosed due to its unusual manifestations in conditions, where the underlying malignancy is not known. PCL as an aggressive malignancy with a very poor prognosis can be presented with STLS signs and symptoms initially. A high level of suspicion is needed for STLS to be diagnosed in early stages to provide a better chance of more immediate and effective treatments, especially in invasive malignancies such as PCL.

## AUTHOR CONTRIBUTIONS

MS contributed in developing the research idea, composing, and revising the manuscript. MI contributed in developing the research idea, composing, and revising the manuscript. MB contributed in composing and revising the manuscript. SP contributed in developing the research idea and revising the manuscript.

## CONFLICT OF INTEREST

The authors have no conflict of interest to declare.

## ETHICAL APPROVAL

This study was approved by the research and ethics committee of Tehran University of Medical Sciences. The patient has given her informed consent to publish this case.

## CONSENT

Written informed consent was obtained from the patient for publication of this case report and any accompanying images. A copy of the written consent is available for review by the Editor‐in‐Chief of this journal.

## Data Availability

Data sharing is not applicable to this article as no datasets were generated or analyzed during the current study.
