# Erratum

**DOI:** 10.1002/ccr3.6271

**Published:** 2022-09-05

In the article entitled “Plasma Cell Leukemia Presenting as Spontaneous Tumor Lysis Syndrome with Hypercalcemia”^1^ that was previously published in Volume 10 Issue 7 of *Clinical Case Reports*, another author's name ‘Narjes Zarei Jalalabadi’ has to be included.

The correct list should be as follows: Mahan Shafie, Mahbod Issaiy, Mahdi Barkhori, Samaneh Parsa, Narjes Zarei Jalalabadi.

The publisher apologizes for the error.
